# Common Pitfalls in using Online Platforms for Data collection in COVID times and its implications

**DOI:** 10.3126/nje.v10i4.31614

**Published:** 2020-12-31

**Authors:** Hariom Kumar Solanki, P. Giridara Gopal, Rama Shankar Rath

**Affiliations:** 1 Department of Community Medicine, Greater Noida Institute of Medical Sciences, Greater Noida, Uttar Pradesh; 2 Centre for Community Medicine, All India Institute of Medical Sciences, New Delhi; 3 All India Institute of Medical Sciences, Gorakhpur, Uttar Pradesh

Sir,

In recent COVID-19 time when the direct physical data collection is quite impossible, we authors came across various online methods of data collection related to various social, behavioral research. We congratulate those researchers who have the burning desire to conduct research and they find various ways to do the same. With this letter we want to highlight few pitfalls related use of online data collection tools.

Online forms allow for remote and easy data collection [1]. Availability of smart phone with internet access on the part of study participants as well as the investigators is sufficient for developing and carrying out data collection for studies based on self-filled questionnaires. In Indian context where many of the studies are done by early career researchers are not supported (non-funded), the cost saved on paper and printing can be considerable [2]. This form of data collection also ensures security of data collected and issues associated with handwriting deciphering.

Recently, there has been spurt in studies being conducted using online forms. In the prevailing situation of COVID19 pandemic where measures like social distancing and avoiding unnecessary travel are important to prevent outbreak exacerbation, it seems to be a pragmatic option given the benefits listed above. However, there are certain pitfalls to doing studies in such a way not meeting certain condition.

First issue pertains to exclusion of people based on their inability to afford, or their unwillingness to use electronic gadgets like smartphone and computer and internet [3]. This will to certain extent make the study participants non-representative of study population which the researcher intended to cover.

Second important issue is fulfillment of condition of probability sampling [3]. In the probability sampling every unit has a known probability of getting selected in the study. However, dissemination of online form for data collection from participants is akin to convenient sampling method where people within the networks of the researcher/s or networks accessible to the researcher/s will be tapped into, especially when there is no clearly defined group of eligible people. Most of the times a request to forward the link to the form is also made by the research team. Some of the participants may circulate it, others may not. Thus, the degree of penetration of the link into the sampling frame may be unknown for many of the studies done through online forms. Both these factors contribute to difficulty in ascertaining the probability of inclusion of sampling units in the study and thus affecting the external validity of the study. In the same way self-selection of individuals is another part which may also lead to selection bias [3,4].

Third issue pertains to the issue of response rate. In absence of knowledge of the number of eligible people reached, the responses rate cannot be ascertained. As explained above it is exceedingly difficult to ascertain the number of eligible people reached in this method especially when the study is not limited to a defined number of institutions with a common communication group [5]. However, issue of higher nonresponse rate in web based and online surveys is not a problem in current scenario of increased use of internet [6].

Similarly, there is an issue related to the responders of such studies. Filling the form multiple times by the same responder/s due to misunderstanding/ mistakes like filling the form again on receiving the link repeatedly with request to fill the form without realizing that the form was meant to be filled only once; may bias the study results [7]. Sometimes some responders may also have strong interest in the academic area study is covering and they might strongly feel that study should favor result in some particular way, this may also motivate some responders to fill the form multiple times or forward the form to individuals in his/ her network who think like them and likely to favor a particular pattern of responses. Both these situations introduce bias into the study which are difficult to identify and control for once the study has been completed. This issue however may be solved in closed group studies, the researchers can limit multiple submission of forms by participants by providing a unique response code to each intended participant in advance which he or she can use only once in the duration of data collection of a particular study. In contrary nonresponse in an internet based or online survey may be due to disease condition studied [8]. Thus, higher and lower response rate in web-based surveys must be considered while interpreting the results.

Another issue related to responders in open study groups arises when the responder is not a part of the intended study population. For example, if a group of researchers want to study an aspect of occupational health in workers of a particular occupational group (say health care workers level of stress in the times of COVID19 pandemic) in a particular administrative unit/ region/ area. It is possible that healthcare workers will have professional links outside that administrative unit/ area and the link is shared with professional working in other administrative units, where the independent/ exposure variable is different from the unit where the study was intended for. The responses registered from outside the intended study population will thus bias the results depending upon multiple independent variables in unknown ways.

We therefore recommend using online form-based study only in well identified group of people preferably in a closed group through a common and secure thread. We also discourage the practice of asking to forward the online link into the contacts and networks of individual study participants except when snowball sampling is being attempted or list of all eligible participants is available from reliable databases. The number of registered medical practitioner in a country or state for example may be available from relevant medical council. When reporting such a study the total number of people who received an online link -whether directly or indirectly - should be incorporated in the body of the report.

In conclusion, though useful when used thoughtfully and for specific well identified groups of individuals, online form based studies might suffer from certain significant infirmities from epidemiological point of view which may cast a shadow of doubt on the reliability and validity of the findings of such studies. This should be kept in mind by both the researchers as well as readers of the research findings/ articles, especially in the prevalent COVID- 19 pandemic situation when researchers are rushing to complete their studies on one or other aspect of COVID-19 and report their findings as soon as possible for publication in academic journals on the one hand and journals are speeding up the process of editorial and peer review for quick dissemination of research findings.

Dr. Hariom Kumar Solanki, Assistant Professor, Department of Community Medicine, Greater Noida Institute of Medical Sciences, Greater Noida, Uttar Pradesh
Dr. P. Giridara Gopal, Scientist C, Centre for Community Medicine, All India Institute of Medical Sciences, New Delhi
Dr. Rama Shankar Rath, Assistant Professor, All India Institute of Medical Sciences, Gorakhpur, Uttar Pradesh

Dated the 28 September 2020
